# Laboratory evaluation of efficacy and persistence of a 1 % *w*/*w* fipronil pour-on formulation (Topline^®^) against *Glossina palpalis gambiensis*, Diptera: Glossinidae

**DOI:** 10.1007/s00436-015-4493-0

**Published:** 2015-05-06

**Authors:** B. Bauer, M. P. O. Baumann

**Affiliations:** Institute for Parasitology and Tropical Veterinary Medicine, Freie Universitaet Berlin, Robert-von-Ostertagstr. 7-13, Berlin, 14163 Germany; FAO Reference Centre for Veterinary Public Health, Faculty of Veterinary Medicine, Freie Universitaet Berlin, Berlin, 14163 Germany

**Keywords:** Flies, *Glossina palpalis gambiensis*, Tick infestation

## Abstract

One zebu bull of 365 kg live weight was treated along the back line with 36 mL of fipronil as a pour-on formulation. Long-lasting mortalities of *Glossina palpalis gambiensis* were recorded despite exposure to sunlight and regular rinsing with 50 L of water during the following 5 months. Significantly higher mortalities were still observed even 140, 170 and 190 days after treatment following their triple releases or triple feeding of caged tsetse on the treated bull. Mortalities of 70, 80 and 44 %, respectively, were recorded after 15 days of observation. This contrasted with the mortalities of control flies that were released in the presence of the untreated bull or fed in cages on the animal, amounting to 20 and twice 10 % after 170 and 190 days. The feeding successes of the released or caged flies were higher than 95 % and did not differ between control and experimental groups, indicating no repulsive or irritant effects of fipronil. The findings of this study are discussed, particularly in view of the potential of fipronil as an effective means for tsetse control.

## Introduction

Treatment of livestock with acaricides is common practice for the control of tick infestations in sub-tropical or tropical climates. With the advent of pyrethroids displaying acaricidal as well as insecticidal effects, this technique has been increasingly used in Sub-Saharan Africa for the simultaneous control of ticks and tsetse flies (Bauer et al. 1988a, 1992a, b, 1995a, b, 1999). In view of an increasing resistance against pyrethroids in ticks, the development and subsequent marketing of fipronil offered a valuable alternative for tick control in countries with important beef industries in Latin America. Fipronil is a phenylpyrazole agent interacting in insects with ligand-gated chloride channels (Hunter et al. 1994), controlled by the neurotransmitter gamma-aminobutyric acid (GABA), thereby blocking pre- and post-synaptic transfer of chloride ions across cell membranes. The blockade leads to an uncontrolled activity of the central nervous system followed by death of exposed ecto-parasites. Presently, fipronil is used against tick infestations of beef cattle in Latin America and, in combination with additional active ingredients, against ecto-parasites of companion animals (Bouhsira et al. 2011). During our study, repeated series of laboratory bio-assays were conducted, evaluating the effects of fipronil against tsetse flies.

## Material and methods

Two male zebu bulls of comparable size and attractiveness were used for the releases of tsetse flies. According to the recommendations of the provider, 36 mL (1 mL/10 kg body weight) of fipronil was applied as a single stripe along the back line of a bull with 365 kg live weight. The treated bull was rinsed with 50 L of water and exposed to sunlight for 3 h every 2nd day during the whole experimental period, thereby taking into account a potentially negative influence of rain and UV radiation on the persistence of the product. Since the study lasted for 190 days, each bull was rinsed on average with 4.750 L of water and exposed to sunlight for 285 h. Males and females of *Glossina palpalis gambiensis* were derived from the tsetse colonies at CIRDES, Bobo-Dioulasso, Burkina Faso, where an independent large-scale in vitro rearing facility had provided sterile males for their subsequent releases in the agro-pastoral zone of Sidéradougou, Burkina Faso (Bauer et al. 1984). All tsetse flies were released from a lock basin into two separate fly-proof facilities with inner dimensions of 2 m in height, 7 m in total length and 3.2 m in width. The assessment took place in several steps: first, 200 flies each were released in the presence of both zebus and re-captured after 2 h. They were then maintained as described (Bauer et al. 1984) on an in vitro feeding system for 15 days in an insectary at 24–26 °C and 80 % relative humidity. This phase lasted for 4.5 months.

Secondly, following the impression of a still higher mortality in comparison to the controls, triple releases were undertaken to assess a potentially persisting mortality 140 days after treatment of the bull.

Thirdly, considering the stress due to re-capture of the released flies and a subsequent higher mortality, it was decided to attach fly cages to the bellies of both bulls allowing the flies to feed on the 1st, 2nd and 6th day after emergence 170 days after treatment. Following their feeding, the tsetse were transferred into new cages and then maintained as explained above.

A concluding feeding trial using caged tsetse took place 190 days after treatment. Again, both experimental and control tsetse were given an opportunity to feed three times on either the treated or the control bull.

Calculations of the 95 % confidence intervals (CIs) were performed for the mean mortality rates.

## Results

### Feeding success

During this study, comparable feeding successes, i.e. exceeding 95 %, irrespective of an eventual effect due to the treatment with fipronil, were recorded for both control and experimental flies. This was contrasting with high proportions of non-engorged tsetse when pyrethroids as deltamethrin or cyfluthrin had been used (Bauer et al. 1992a, 1995a).

### Single releases

**Fig. 1:**
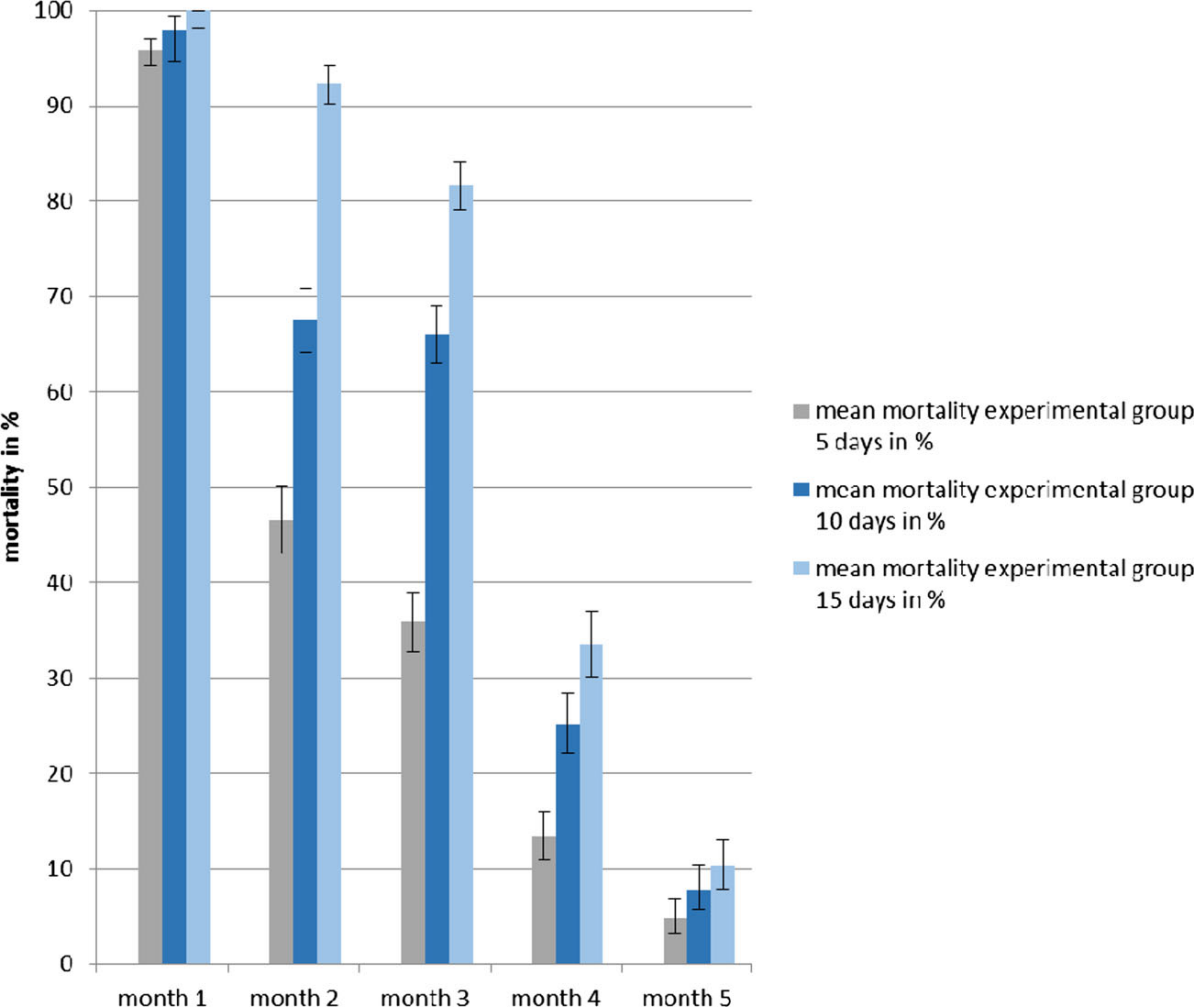
Mean mortalities during 5 months (with 95 % confidence intervals) of the released *G. p. gambiensis* at days 5, 10 and 15 feeding on a fipronil treated bull

**Fig. 2:**
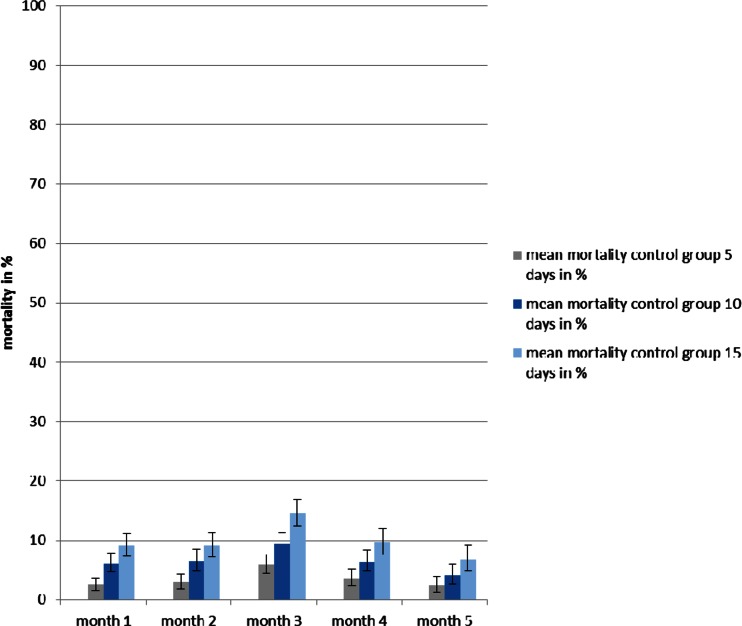
Mean mortalities during 5 months (with 95 % confidence intervals) of the released *G. p. gambiensis* at days 5, 10 and 15 feeding on an untreated bull

All fipronil-exposed flies died during the 1st month within the 15 following days. At the same time, only 5–10 % of the released flies had died in the control groups. While more than 90 % of all the flies died during the first 5 days in the 1st month, a shift in the effect of fipronil was observed during the two subsequent months. Less than 50 % of the overall mortality was recorded after 5 days, but the remaining, larger proportion of an average mortality of 80–90 % was noted during days 6 to 10. At the same time, the comparative difference of mean mortalities remained highly significant (*p* < 0.001) between tsetse released in the presence of the treated bull and their controls. During the 4th month, the mortality decreased to 33.6 %, yet this difference was still significant (*p* < 0.01) when compared to a mean mortality of 9.7 % of the control flies. Single releases were terminated during the 5th month with average mortalities of 10.3 and 6.9 % for experimental and control flies, respectively (Figs. 1 and 2).

### Triple releases 140 days after treatment

Repeated release and re-capture of flies is likely to result in stress and subsequent higher mortalities. Indeed, the mortality of the control group (20 %) was higher than the usual probably due to the triple releases/re-captures. Yet, the corresponding mortality for the experimental flies amounted to 70 %.

### Triple feeding of caged tsetse 170 days after treatment

**Fig. 3:**
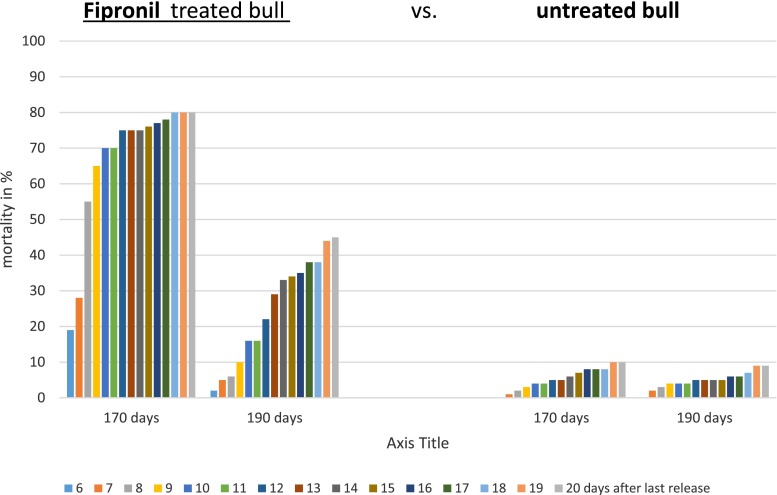
Mean mortalities of the caged *G. p. gambiensis* after triple feeding on a fipronil treated bull vs. an untreated bull 170 and 190 days after treatment

Triple feeding of the caged tsetse 170 days after treatment resulted in approximately 80 % mortality of the flies when they were fed three times on the belly of the treated bull compared to around 10 % mortality of the control flies (Fig. 3).

### Triple feeding of caged tsetse 190 days after treatment

A final assessment—still indicating persistent differences—after 190 days was again based on triple feeding. Nearly 44 % of the experimental flies died within 15 days, while only around 10 % mortality was observed in the control group (Fig. 3).

## Discussion

Based on the behaviour of tsetse and other arthropods landing and feeding on distal body parts, a homogeneous dispersal of the active ingredient would be highly desirable. While pour-on formulations of pyrethroids have shown considerable potential in controlling tsetse and other ecto-parasites (Bauer et al. 1989, 1992b, 1995b, 1999), dispersal studies with the caged tsetse have shown persisting maximum residual effects mostly along the dorsal line of application. However, with increasing time and distance from the dorsal line, the effects of many products diminished (Bauer et al. 1992a; 1995b). Elsewhere, pyrethroids as spray or pour-on formulations have been successfully used for the control of other haematophagous insects, particularly biting midges during the outbreak of bluetongue virus infections in Europe (Mullens 1993, 2001, 2010; Doherty et al. 2001; Mehlhorn et al. 2008a; Liebisch et al. 2008b; Papadopoulos et al. 2009; Schmahl et al. 2009). However, Stendel et al. (1992) have demonstrated a suboptimal dispersal of topically applied flumethrin with highest concentrations of the active ingredient along the dorsal line, whereas belly and limbs showed lowest concentrations. Poor dispersal of the active ingredient may result in suboptimal control of many arthropods (Bauer et al. 1995). Mullens et al. (2010) tried to overcome this problem by treating exposed parts of the body such as face, ears and belly of sheep to protect them against biting midges. The adult West African cattle tick *Amblyomma variegatum* has its primary fixation site near the claws of cattle as its preferential host (Stachurski 2000). Considering its vectorial competence in transmitting cowdriosis and dermatophilosis as well as its role in causing cutaneous lesions, an effective control of *Amblyomma* spp. is of high priority for cattle owners in much of Sub-Saharan Africa. High costs for many products, and the fact that there is a need for repeated treatments during the rainy season, may add to the reservations against a large-scale adoption of using pour-on formulations of pyrethroids. Communal dipping schemes proved to be effective but were not maintained in the absence of external funding and proper management. The introduction of targeted treatments of the predilection sites reduced costs and also addressed the issue of arthropods’ feeding preferences (Vale 2003; Torr et al. 2007). The advent of foot baths (Stachurski 2000; Stachurski and Lancelot 2006; Bouyer et al. 2007, 2009) has helped to reduce costs and time required for treatments, but questions remain about the sustainability (Bouyer et al. 2011). Infrastructural needs may be less for foot baths, yet the construction of a foot bath and a cattle crush is still necessary as well as the continuous surveillance of dip strength and safe disposal of dip remnants. Hardship of regular treatments at fortnightly intervals, particularly during the rainy season, has been discussed elsewhere (Davey et al. 1998). Continuous training and follow-up by the communities may thus be required before foot baths can be considered as a sustainable method for the prevailing extensive husbandry management systems (Bouyer et al. 2011).

The present study has shown an exceptional persistence of fipronil against tsetse, distinctly exceeding the most persistent deltamethrin pour-on formulations. Significantly higher mortalities were recorded during 4 months of single releases. Even 140 days after treatment, triple releases of tsetse resulted in a mortality of 70 %, which was significantly higher compared to control groups with 20 % mortality. Fipronil could offer a valuable, cost-effective alternative for the control of tsetse and other arthropods in extensive animal husbandry management systems, not requiring investments in infrastructure. Its long-lasting effects might allow a treatment interval of 5–6 months for the control of tsetse. Fipronil does not prevent tsetse from feeding, contrary to some pyrethroid formulations (Bauer et al. 1992a; 1995b). As a consequence, the transmission of trypanosomes may continue, leading to a comparatively slower decrease of trypanosomosis incidence during a tsetse control campaign. Logan et al. (2010) have suggested applying principles of a ‘push-pull’ strategy for the control of ecto-parasites. In our case, valuable pyrethroid-treated livestock might irritate alighting insects and prevent them from feeding, while young or unproductive stock would benefit from a fipronil treatment. Fipronil is described as a non-systemic acaricide, although a withdrawal period of 100 days for treated beef cattle is recommended by the manufacturer. Since the feeding success of tsetse remained close to 100 %, it appeared that high mortalities were linked to feeding success. In another work on the effects and persistence of fipronil against *Boophilus microplus* (Davey et al. 1998), it was concluded that the ticks needed to engorge on their host before impacts of the treatment could be expected. Additional studies on the mechanisms of potential systemic effects and how to minimize eventual consumers’ risks are therefore recommended.
